# Phasing out the bad—How SQSTM1/p62 sequesters ubiquitinated proteins for degradation by autophagy

**DOI:** 10.1080/15548627.2018.1462079

**Published:** 2018-07-20

**Authors:** Gabriele Zaffagnini, Adriana Savova, Alberto Danieli, Julia Romanov, Shirley Tremel, Michael Ebner, Thomas Peterbauer, Martin Sztacho, Riccardo Trapannone, Abul K. Tarafder, Carsten Sachse, Sascha Martens

**Affiliations:** a Department of Biochemistry and Cell Biology, Max F. Perutz Laboratories (MFPL), Vienna Biocenter (VBC), University of Vienna, Vienna, Austria; b Structural and Computational Biology Unit, European Molecular Biology Laboratory, Heidelberg, Germany; c Department of Structural and Computational Biology, Max F. Perutz Laboratories (MFPL), Vienna Biocenter (VBC), University of Vienna, Vienna, Austria

**Keywords:** Selective autophagy, aggrephagy, cargo receptor, ubiquitin, p62, phase transition, quality control, LC3

## Abstract

The degradation of misfolded, ubiquitinated proteins is essential for cellular homeostasis. These proteins are primarily degraded by the ubiquitin-proteasome system (UPS) and macroautophagy/autophagy serves as a backup mechanism when the UPS is overloaded. How autophagy and the UPS are coordinated is not fully understood. During the autophagy of misfolded, ubiquitinated proteins, referred to as aggrephagy, substrate proteins are clustered into larger structures in a SQSTM1/p62-dependent manner before they are sequestered by phagophores, the precursors to autophagosomes. We have recently shown that SQSTM1/p62 and ubiquitinated proteins spontaneously phase separate into micrometer-sized clusters *in vitro*. This enabled us to characterize the properties of the ubiquitin-positive substrates that are necessary for the SQSTM1/p62-mediated cluster formation. Our results suggest that aggrephagy is triggered by the accumulation of substrates with multiple ubiquitin chains and that the process can be inhibited by active proteasomes.

The accumulation of misfolded proteins is a particularly dangerous threat for the cell, and multiple systems have evolved to identify and, if necessary, degrade these proteins. In the ubiquitin-proteasome system (UPS), misfolded proteins become tagged with ubiquitin chains. They are subsequently recognized by proteasomal receptors where the ubiquitin chains are removed, the substrate is unfolded and finally degraded. When the UPS is overloaded, autophagy serves as an important backup system by clustering the ubiquitin-positive proteins into larger structures that become enclosed within autophagosomes and subsequently degraded within lysosomes. This process is also referred to as aggrephagy. The autophagy cargo receptor SQSTM1/p62 plays an important role in aggrephagy as it links the cargo material to the nascent phagophore membrane due to its ability to bind to ubiquitin and Atg8-family proteins that decorate the membrane. SQSTM1/p62 is also important for the clustering of substrate proteins into larger structures upstream of autophagosome formation.

How the UPS and autophagy are coordinated is largely unclear, mainly due to the lack of knowledge about the nature of the substrates that trigger SQSTM1/p62-dependent aggrephagy. In a reconstituted *in vitro* system, we have recently shown that SQSTM1/p62 and certain ubiquitinated proteins spontaneously phase separate into micrometer-sized clusters. This allowed us to conduct a detailed characterization of the properties of SQSTM1/p62 and the substrates required to trigger phase separation. We found that the reaction depends on the ability of p62 to self-assemble via its N-terminal PB1 domain and to bind ubiquitin via its C-terminal UBA domain. Phase-separation activity is enhanced by a phospho-mimicking mutation in the UBA domain that facilitates ubiquitin binding and can be stimulated by the NBR1 cargo receptor. We further found that phase separation is very sensitive to the concentration of the ubiquitinated substrates and to the length of the ubiquitin chains. In particular, we discovered that the best substrates in our reconstituted system have at least 2 ubiquitin chains with more than 3 ubiquitins attached to it. Free ubiquitin chains of 4 ubiquitins or single ubiquitin chains with 4 ubiquitins attached to a substrate do not trigger clustering. Longer chains, however, may also trigger cluster formation in our system. While we conducted the initial experiments with linear (M1-linked) ubiquitin chains, the physiologically relevant substrates are likely to harbor K48- and/or K63-linked ubiquitin chains. Therefore, we conjugated these chains to a model substrate protein, revealing that these chain types are also able to support phase separation. Interestingly, K48-linked chains appear to be the least efficient in triggering cluster formation. Since these are the main targets for the proteasome, this implies that they need to accumulate above a relatively high threshold before SQSTM1/p62 can cluster them for autophagy. In addition, we found that free K48-, and to a lesser extent K63-linked chains, inhibit phase separation. This effect is mediated by a previously unknown ubiquitin binding activity of the zinc finger domain of SQSTM1/p62, which may negatively affect oligomerization of SQSTM1/p62 itself. We also found that high concentrations of free mono-ubiquitin inhibit cluster formation. Because ubiquitin chains are thought to be released en bloc by active proteasomes and subsequently hydrolyzed to individual ubiquitins, these findings may suggest that the activity of SQSTM1/p62, and by implication that of aggrephagy, can be coordinated with proteasomal activity.

We then went on to study the properties of the clusters resulting from the phase separation reaction. When we conducted fluorescence recovery after photobleaching (FRAP) experiments we found that the ubiquitinated substrates display fast recovery while the recovery of SQSTM1/p62 is very slow, implying that the substrates can move freely within the clusters while SQSTM1/p62 is rather immobile. The low mobility of SQSTM1/p62 was also supported by structured illumination microscopy experiments conducted with 2 differently fluorescently labelled SQSTM1/p62 proteins. When we performed FRAP experiments with human cells expressing an endogenously GFP-tagged SQSTM1/p62 protein, we also observed low recovery of the protein in cellular puncta. We then employed negative stain electron microscopy to elucidate the structural basis for the cluster formation. It has previously been shown that purified SQSTM1/p62 exists as helical filaments and we found that these filaments coalesce in the presence of a ubiquitinated substrate, suggesting that the substrates crosslink the filaments (Figure 1). Supporting the physiological relevance of this model, we found by fluorescence correlation spectroscopy using the cells expressing the endogenously GFP-tagged SQSTM1/p62, that the protein mainly exists as homo-oligomers in these cells.

**Figure 1.**
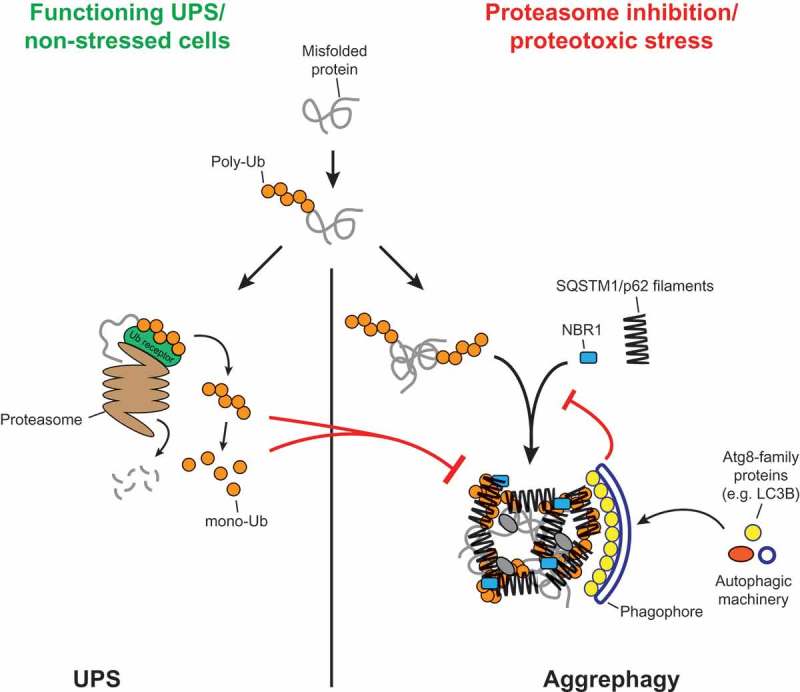
Model for the crosslinking of SQSTM1/p62 filaments by ubiquitinated proteins, and the coordination of this process with the activity of the UPS and the autophagy machinery. The figure was taken from Zaffagnini et al., EMBO J, 2018, doi: 10.15252/embj.201798308 with permission of the publisher.

We finally asked if the *in vitro* formed clusters containing SQSTM1/p62 and the ubiquitin-positive substrates are able to recruit Atg8-family proteins. To this end, we added LC3B to the clusters and found that it is readily recruited to the clusters. Interestingly, we found that the presence of LC3B inhibits the clustering reaction to some extent. LC3B binds to the LIR motif of SQSTM1/p62, which is situated in an intrinsically disordered region of the protein. In order to test if the inhibition by LC3B is caused solely by a steric effect or if the LIR motif also directly contributes to the clustering activity of SQSTM1/p62, we tested a LIR mutant version of SQSTM1/p62 and found that this mutant shows severely reduced cluster formation. Consistently, when we introduced the LIR mutation into the endogenous GFP-tagged SQSTM1/p62 protein in human cells, we found reduced puncta formation. These results suggest that the cluster reaction might be coordinated with the formation of the phagophore membrane, on which LC3B is concentrated (Figure 1).

In summary, our study shows that SQSTM1/p62 is fully sufficient to phase separate ubiquitinated proteins into larger structures and suggests how this process can be coordinated with the activity of the UPS and autophagosome formation (Figure 1).

